# Criteria for the Transition to Adulthood, Developmental Features of Emerging Adulthood, and Views of the Future Among Greek Studying Youth

**DOI:** 10.5964/ejop.v13i3.1327

**Published:** 2017-08-31

**Authors:** Evangelia Galanaki, Sophie Leontopoulou

**Affiliations:** a Department of Primary Education, School of Education, National and Kapodistrian University of Athens, Athens, Greece; b Department of Primary Education, School of Education, University of Ioannina, Ioannina, Greece; Webster University Geneva, Geneva, Switzerland

**Keywords:** emerging adulthood, transition to adulthood, optimism, studying youth, Greece

## Abstract

This study investigated emerging adulthood and transition to adulthood in Greece, a highly underresearched issue in this country. Participants were 784 university students aged 17.5-27.5 years. Criteria for the transition to adulthood, developmental features of emerging adulthood, perceived adult status, views of the future (optimism), and sociodemographic variables were assessed. The results support the existence of emerging adulthood as a distinct life period in Greece. More than two thirds of the sample were self-perceived emerging adults. Most prevalent criteria were Norm compliance and Family capacities. Developmental features of emerging adulthood ranked high, especially Identity exploration, Experimentation/possibilities, and Feeling “in-between”. Statistically significant variations emerged as a function of gender, age, living arrangement, job experience, and perceived adult status. Views of the future were cautiously optimistic. Similarities with existing data and differences related to the specific characteristics of the Southern European context are discussed.

Although Erikson (1950, 1968) has argued that young people in industrialized societies continue to explore their identity well beyond adolescence, it is only recently that emerging adulthood was proposed as a distinct developmental period from the late teens to the late twenties (ages 18-29) (Arnett, 2000a). According to this conceptualization, emerging adulthood is characterized by (a) identity exploration in love, work and worldviews; (b) active experimentation, many possibilities, open choices and optimism; (c) a sense of negativity and instability due mainly to the unstructured nature of this period and the many changes taking place in several life domains; (d) increased self-focus with the aim to attain self-knowledge and self-sufficiency, facilitated by the absence of obligations, and (e) a feeling “in-between” adolescence and adulthood, that is an ambivalence toward adult status.

Over the past two decades, emerging adulthood has been investigated in several parts of the world, such as the USA, Europe, Japan, China, India, etc. In post-industrial societies, based on knowledge, technology, and services, and under the influence of globalization, education is prolonged in order to lead to a better occupational status, and transitions to job, marriage and parenthood are postponed, therefore there is a rather long route to adulthood. Although considerable criticism has been raised against the universality of emerging adulthood and, therefore, its existence as a developmental stage, a useful research aim is to evaluate for whom, under what conditions and to what degree variations in the experience of emerging adulthood appear within each culture (Arnett, 2011; Syed, 2016).

In Europe, a body of recent research has showed culturally-determined similarities and differences in the pathways to adult life (Žukauskienė, 2015). As far as the Mediterranean countries are concerned, strong family ties and solidarity, as well as high unemployment and underemployment rates, restricted social welfare, and other financial difficulties have led to prolonged co-residence with parents and late marriage and parenthood (Iacovou, 2002, 2010). This has been described as the Mediterranean or the Southern European model (Ferrera, 1996; Scabini, 2000).

Sociodemographic shifts in Greece have possibly contributed to the rise of emerging adulthood in this country (Eurostat, 2016). The percentage of young people aged 20-29 who live with their parents is 71.5% (one of the highest in Europe), and the age of leaving the parental home is 29.3 years (30.6 for males, 28.0 for females). Age at first marriage is 32.9 for males and 29.7 for females. Mean age of women at first childbirth is 31.1 (one of the highest in Europe). Unemployment among 15-24 year-olds reaches 51.9% (also the highest in Europe). The percentage of 18 year-olds attending tertiary education is also the highest: 47.2%. Rather high is the median age of students in tertiary education: 23.9 years. The employment rate of recent graduates is the second lowest in Europe: 44.3%.

Surprisingly, thus far only two studies in Greece have investigated the criteria for the transition to adulthood (Petrogiannis, 2011) and the developmental features of emerging adulthood (Leontopoulou, Mavridis, & Giotsa, 2016). Sociodemographic changes, in combination with the 2008 financial crisis which has severely affected Greece, dictate the need to extend relevant research in this country.

## Feeling “in-between”

A defining feature of emerging adulthood is the feeling of young people that they are on the way to adulthood but not there yet. Many young people are often ambivalent about their status as adults. They respond “in some respects yes, in some respects no” when asked whether they have reached adulthood, a finding that supports the existence of emerging adulthood.

Feeling “in-between” is most prevalent (exhibited by about two thirds of youth) in Western cultures, such as the USA (Arnett, 2001, 2003; Badger, Nelson, & McNamara Barry, 2006; Nelson & Barry, 2005), Austria (Sirsch, Dreher, Mayr, & Willinger, 2009), Sweden (Wängqvist & Frisén, 2015), and Denmark (Arnett & Padilla-Walker, 2015). High percentages of this feeling were also found in Greece (Petrogiannis, 2011), Turkey (Doğan, Yüzbaşi, & Demir, 2015), Czech Republic (Macek, Bejček, & Vaníčková, 2007), Romania (Nelson, 2009), Argentina (Facio & Micocci, 2003), and Aborigins of Australia (Cheah & Nelson, 2004). The lowest percentages were found in China (Nelson, Badger, & Wu, 2004), India (Seiter & Nelson, 2011), and in ethnic minorities (Arnett, 2003; Badger et al., 2006; for a more extensive review, see Nelson & Luster, 2015). It appears, then, that emerging adulthood is more prominent in Western and Westernized cultures, but is not absent in quite different cultures.

## Criteria for the Transition to Adulthood

In this study, a variety of perceived criteria for the transition to adulthood are examined, on the basis of the model proposed by Arnett (2001). It includes criteria drawn from biology, anthropology, sociology, law, and psychology.

Research has shown (Nelson & Luster, 2015, for a more extensive review) that emerging adults – especially university students – in Western cultures prioritize Independence criteria, such as responsibility for one’s actions, independent decision making, equality with parents, and financial independence. This finding is common in the USA (Arnett, 2001, 2003), Austria (Sirsch et al., 2009), Sweden (Wängqvist & Frisén, 2015), Denmark (Arnett & Padilla-Walker, 2015), and Israel (Mayseless & Scharf, 2003). A different picture has emerged in rather collectivistic cultures, namely China (Badger et al., 2006; Nelson et al., 2004; Nelson, Duan, Padilla-Walker, & Luster, 2012; Zhong & Arnett, 2014), and India (Seiter & Nelson, 2011), where communal criteria, such as Relational maturity (e.g., accepting responsibility for one’s actions *and* having emotional control), were more important. A mixture of individualistic and collectivistic criteria for the transition to adulthood is present in Eastern European countries such as Lithuania (Vosylis, Kaniušonytė, & Raižienė, 2015) and Romania (Nelson, 2009), as well as Turkey (Doğan et al., 2015), and Argentina (Facio & Micocci, 2003). Similar findings are typical of Southern European countries, namely Italy (Crocetti & Tagliabue, 2015; Piumatti, Garro, Pipitone, Di Vita, & Rabaglietti, 2016) and Greece (Petrogiannis, 2011); in the latter study, Independence, Family capacities, and Norm compliance emerged as the key markers of adulthood.

## Developmental Features of Emerging Adulthood

Research into perceived developmental features of emerging adults has been conducted with the *Inventory of the Dimensions of Emerging Adulthood* (IDEA; Reifman, Arnett, & Colwell, 2007), more frequently in university students. Emerging adulthood appeared to be mainly a time of Identity exploration and Experimentation/possibilities in Austria (Sirsch et al., 2009), Greece (Leontopoulou et al., 2016), and Romania (Negru, 2012). In Italy, Identity exploration and Self-focus were the highest, whereas in Japan, Experimentation/possibilities scored the highest (Crocetti et al., 2015). Experimentation/possibilities and Self-focus were the main features of youth in Turkey (Atak & Çok, 2008), followed by Negativity/instability, Identity exploration, and Feeling “in-between”. A somewhat different picture emerged in Germany (Seiffge-Krenke, 2015), where Negativity/instability and Self-focus were highly prevalent. Similar findings emerged with adaptations of the IDEA in Czech Republic (Macek et al., 2007) and among Mexican and Spanish youth (Fierro Arias & Moreno Hernández, 2007). It is evident that there is not enough research on the above developmental features of emerging adulthood all over the world.

## Age of Optimism

A central feature of emerging adulthood is optimism (Arnett, 2000a). Young people feel that they have many open choices and possibilities, perhaps more than in any other life period. Emerging adults in various cultures feel optimistic about their quality of life, financial well-being, career achievements, and personal relationships in the future (Arnett, 2000b; Nelson, 2009; Nelson et al., 2004; Seiter & Nelson, 2011). More research is needed on this issue too across cultures.

## Current Study

This study focuses on emerging adulthood and transition to adulthood in Greece. The following issues are examined: (a) Perceived adult status in relation to specific sociodemographic variables (i.e., gender, age, father’s education, living arrangement, and job experience); (b) the criteria which signify the transition to adulthood, according to young people’s views; (c) the extent to which young people experience the defining features of emerging adulthood; (d) sociodemographic variables and perceived adult status in relation to transition criteria and perceived developmental features; and (e) young people’s optimism about the future. Up to date, there is very limited research evidence, within the theoretical framework of emerging adulthood, regarding these issues in Greece. Moreover, there are no data available in the Greek context for the factorial structure of the instruments measuring the criteria for adult status and the developmental features of emerging adulthood (with only one exception: Leontopoulou et al., 2016). These issues are examined in a large and representative sample of Greek university students. We should note here that, in Greece, about half of young people are enrolled in tertiary education, which is free but requires rigorous entrance examinations. Preparation for these examinations starts early (from the last two years of secondary education) and there is minimum flexibility and mobility during years of study, once the entrance in a university department is achieved.

## Method

### Participants

Participants were 784 undergraduate students who were recruited during scheduled class hours from various departments (i.e., Education, Psychology, Physics, Economy) of the National and Kapodistrian University of Athens, Athens, Greece, and voluntarily agreed to take part in the study. Participation rate was over 90%. Mean age was 20.0 years (*SD* = 1.85, range 17.5-27.5 years), and 62% were women. Table 1 presents sociodemographic characteristics of the sample. As can be seen, the vast majority were single, with no children, and had fathers with at least medium educational level (secondary and tertiary education). A small percentage of students (11.5%) had full-time job experience, and somewhat less than 50% had part-time job experience. There were no significant differences between the two types of living arrangement (i.e., living with parents and living without parents) as a function of job experience, χ^2^(2, *N* = 779) = 3.22, *ns*. A very small percentage came from other countries (Cyprus: 1.5%; Eastern European Countries: 2.8%).

**Table 1:** Sociodemographic Characteristics of the Sample (N = 784)

Variable	*f*	%
Gender		
Men	298	38.0
Women	486	62.0
Country of origin^a^		
Greece	749	95.5
Other	34	4.3
Father’s education^a^		
Low^b^	126	16
Medium^b^	332	42.4
High^b^	325	41.5
Living arrangement^c^		
With parents	437	55.7
Without parents	342	43.6
Marital status^a,d^		
Single	770	98.2
Engaged	10	1.3
Married	3	0.4
Children		
No	780	99.5
Yes	4	0.5
Job experience		
None	367	46.8
Part-time	327	41.7
Full-time	90	11.5

*Note.* ^a^missing case: 1. ^b^low: up to lower secondary school, medium: up to post-secondary education, high: up to tertiary education. ^c^missing cases: 5. ^d^zero frequencies for divorced and separated status.

### Measures

#### Perceived Adult Status (Arnett, 2001)

Participants were asked “Do you think you have reached adulthood?” (*No* = 0, *In some respects yes, in some respects no* = 1, *Yes* = 2).

#### Scale of Conceptions of the Transition to Adulthood (Arnett, 2001, 2003)

This scale consists of 39 items representing criteria for the transition to adulthood in seven subscales, the following: Independence (e.g., being financially independent from parents); Interdependence (e.g., committed to long-term love relationship); Role transitions (e.g., married); Norm compliance (e.g., avoid drunk driving); Biological transitions (e.g., if a woman, become biologically capable of bearing children); Legal/chronological transitions (e.g., reached age twenty-one); and Family capacities (e.g., if a man, become capable of supporting a family financially). Participants were asked to “indicate whether you think the following must be achieved before a person can be considered to be an adult” (*Necessary for adulthood* = 1, *Not necessary for adulthood* = 0).

The seven subscales have been used in many studies across cultures, without empirical examination of the factorial structure (Arnett, 2001, 2003; Cheah & Nelson, 2004; Cheah, Trinder, & Gokavi, 2010; Crocetti & Tagliabue, 2015; Facio & Micocci, 2003; Kins & Beyers, 2010; McNamara Barry & Nelson, 2005; Nelson & Barry, 2005; Nelson et al., 2004; Petrogiannis, 2011; Piumatti et al., 2016; Sirsch et al., 2009; Wängqvist & Frisén, 2015), whereas this structure was examined in fewer investigations (Arnett & Padilla-Walker, 2015; Badger et al., 2006; Doğan et al., 2015; Mayseless & Scharf, 2003; Tagliabue, Crocetti, & Lanz, 2016). In most of the former studies, some subscales (e.g., Independence) had low Cronbach alphas, whereas in the latter studies, variations from the original theoretical structure of the measure emerged but still some subscales exhibited low reliabilities.

In light of the absence of validity data on this scale in Greece, we performed exploratory factor analysis (EFA) – principal component analysis with varimax rotation – which led to the exclusion of eight items with low loadings (cut-off point: .30). As can be seen in Table 2, the excluded items belong to Independence and Interdependence. The most acceptable and meaningful solution for the remaining items emerged from a principal component analysis with varimax rotation and six fixed factors. This solution explained 42.78% of the total variance, with Kaiser-Meyer-Olkin measure of sampling adequacy .73 and Bartlett’s test of sphericity χ^2^(465) = 4,652.004, *p* < .001. The factors were named Family capacities, Norm compliance, Financial independence, Family formation, Biological parenthood, and Age-related/biological transitions. Cronbach alpha of the total scale was .73 and of the factors .59-.90.

**Table 2:** Exploratory Factor Analysis and Endorsement of the Criteria for the Transition to Adulthood

Factor	Loading	Eigenvalue	% of Variance	Cronbach alpha	%^a^
*Family capacities* (Factor 1)		3.98	12.83	.75	
If a woman, become capable of supporting a family financially	.71				52.2
If a woman, become capable of caring for children	.71				69.0
If a man, become capable of keeping a family physically safe	.66				64.0
If a man, become capable of supporting a family financially	.65				68.3
If a woman, become capable of keeping a family physically safe	.63				48.1
If a man, become capable of caring for children	.54				39.4
If a woman, become capable of running a household	.36				28.7
*Norm compliance* (Factor 2)		2.61	8.40	.72	
Drive safely and close to speed limit	.68				74.8
Avoid illegal drugs	.68				80.2
Avoid becoming drunk	.66				68.9
Avoid committing petty crimes like vandalism and shoplifting	.65				76.9
Avoid drunk driving	.59				87.6
Use contraception if sexually active and not trying to conceive a child	.48				83.3
Avoid use of profanity/vulgar language	.46				36.9
Have no more than one sexual partner	.36				32.5
*Financial independence* (Factor 3)		2.06	6.65	.68	
Become employed full time	.66				32.0
Settle into a long-term career	.61				66.9
Financially independent from parents	.58				63.3
No longer living in parents’ household	.52				31.5
Finish education	.51				29.9
*Family formation* (Factor 4)		1.88	6.06	.63	
Married	.73				4.5
Have at least one child	.72				3.2
Purchase house	.56				10.2
Committed to long-term love relationship	.54				15.7
*Biological parenthood* (Factor 5)		1.45	4.67	.90	
If a man, become biologically capable of bearing children	.92				17.5
If a woman, become biologically capable of bearing children	.92				18.5
*Age-related/biological transitions* (Factor 6)		1.29	4.16	.59	
Grow to full height	.64				62.1
Reached age eighteen	.52				40.9
Reached age twenty-one	.40				19.3
Have had sexual intercourse	.36				57.6
Have obtained driver’s license and can drive an automobile	.36				10.0
*Items with loadings < .30 (excluded)*					
Accept responsibility for the consequences of your action					
Decide on personal beliefs and values independently of parents or other influences					
If a man, become capable of running a household					
Establish a relationship with parents as an equal adult					
Learn always to have good control over your emotions					
Become less self-oriented, develop greater consideration for others					
Make life-long commitments to others					
Not deeply tied to parents emotionally					

*Note.* Percentage (%) of participants answering *yes* to *necessary for adulthood*.

#### Inventory of the Dimensions of Emerging Adulthood (Reifman et al., 2007)

It consists of 31 items assessing developmental characteristics of emerging adulthood in six subscales, which include the five defining features of emerging adulthood and the Other-focus subscale to enable the comparison with the Self-focus subscale: Identity exploration (e.g., time of finding out who you are); Experimentation/possibilities (e.g., time of trying out new things; time of open choices); Negativity/instability (e.g., time of feeling stressed out; time of confusion); Other-focus (e.g., time of commitments to others), Self-focus (e.g., time of focusing on yourself), and Feeling “in-between” (e.g., time of feeling adult in some ways but not others). Participants were asked “Is this period of your life a time of …” (*Strongly Disagree* = 1 to *Strongly Agree* = 4).

An exploratory factor analysis – principal component analysis with varimax rotation – led to the exclusion of three items with low loadings (cut-off point: .30). As can be seen in Table 3, the excluded items belong to Negativity/instability and Self-focus. The most acceptable and meaningful solution for the remaining items emerged from a principal component analysis with varimax rotation and six fixed factors. This solution explained 50.60% of the total variance, with Kaiser-Meyer-Olkin measure of sampling adequacy .78 and Bartlett’s test of sphericity χ^2^(378) = 4,970.859, *p* < .001. The factors were highly similar to those of the original measure, therefore their names were retained. However, there was a small number of deviations from the original structure apart from the three excluded items. Namely, “time of separating from parents” loaded on Self-focus and not on Identity exploration, “time of optimism” loaded on Negativity/instability and not on Self-focus, and “time of feeling restricted” loaded on Self-focus and not on Negativity/instability. Cronbach alpha of the total scale was .74 and of the factors .64-.75.

**Table 3:** Exploratory Factor Analysis and Endorsement of the Developmental Features of Emerging Adulthood

Factor	Loading	Eigenvalue	% of Variance	Cronbach alpha	%^a^
*Identity exploration* (Factor 1)		4.31	15.41	.75	
time of defining yourself	.72				90.2
time of seeking a sense of meaning	.72				83.6
time of finding out who you are	.66				78.4
time of deciding on your own beliefs and values	.63				84.7
time of planning for the future	.46				93.6
time of learning to think for yourself	.45				83.4
*Negativity/instability* (Factor 2)		3.03	10.81	.74	
time of confusion	.73				41.0
time of feeling stressed out	.73				43.0
time of high pressure	.73				56.0
time of instability	.68				59.8
time of optimism	.51				78.8
*Self-focus* (Factor 3)		2.24	7.99	.68	
time of personal freedom	.77				85.7
time of independence	.72				86.5
time of feeling restricted	.63				84.3
time of responsibility for yourself	.52				95.9
time of open choices	.50				82.6
time of separating from parents	.45				69.0
time of self-sufficiency	.43				44.4
*Experimentation/possibilities* (Factor 4)		1.79	6.40	.65	
time of exploration	.74				90.1
time of experimentation	.68				78.0
time of many possibilities	.65				90.0
time of trying out new things	.53				87.7
*Feeling “in-between”* (Factor 5)		1.57	5.61	.75	
time of feeling adult in some ways but not others	.83				83.3
time of gradually becoming an adult	.77				82.4
time of being not sure whether you have reached full adulthood	.75				74.5
*Other-focus* (Factor 6)		1.23	4.39	.64	
time of responsibility for others	.67				50.0
time of settling down	.53				63.5
time of commitments to others	.51				51.5
*Items with loadings < .30 (excluded)*					
time of unpredictability					
time of many worries					
time of focusing on yourself					

*Note.* ^a^*Agree* and *Strongly agree* combined.

#### Views of the Future

Participants were asked the following questions in order to examine their optimism about the future (Arnett, 2000b): “Overall, do you think the quality of your life is likely to be better or worse than your parents’ has been?”; “Overall, do you think your financial well-being in adulthood is likely to be better or worse than your parents’ has been?”; “Overall, do you think your career achievements are likely to be better or worse than your parents’ have been?”; “Overall, do you think your personal relationships are likely to be better or worse than your parents’ have been?” (response options: “Better”, “About the same”, and “Worse”).

The Greek versions of the measures were translated into Greek and back translated into English by two bilingual translators.

### Procedure

Data were collected in group sessions during class hours, after permission from university professors had been granted. The questionnaires were anonymous. Data collection lasted about 30 minutes. Students received no credit for their participation. The research complies with all ethical guidelines set by the Department of Primary Education (following the standards outlined by the Ethics Committee of the National and Kapodistrian University of Athens).

## Results

### Perceived Adult Status

When asked whether they believed they had reached adulthood, more than two thirds of the participants (560; 71.4%) responded “in some respects yes, in some respects no” (emerging adults), 122 (15.6%) responded “yes” (adults), and 102 (13%) responded “no” (non-adults). As shown in Table 4, emerging adults were more likely to be women, from younger age groups, and with no job experience. In contrast, older age groups and students with full-time job experience were more likely to perceive themselves as adults; and men were more likely to perceive themselves as non-adults. There were no significant differences among categories of perceived adult status as a function of father’s education and living arrangement.

**Table 4:** Differences Among Subgroups as a Function of Perceived Adult Status

Variable	Perceived Adult Status						*p* (χ^2^ test)		
	Non-adult		Emerging adult		Adult		Non-adult	Emerging adult	Adult
	*f*	*%*	*f*	*%*	*f*	*%*			
Gender							.000	.000	.253
Men	57	19.1	189	63.4	52	17.4			
Women	45	9.3	371	76.3	70	14.4			
Age group							.237	.000	.000
17.5-19.9	59	12.4	372	78.0	46	9.6			
20-21.9	22	11.6	131	69.3	36	19.0			
22-27.5	21	17.8	57	48.3	40	33.9			
Father’s education							.641	.547	.813
Low	18	14.3	86	68.3	22	17.5			
Medium	46	13.9	235	70.8	51	15.4			
High	38	11.7	238	73.2	49	15.1			
Living arrangements							.552	.997	.580
With parents	60	13.7	313	71.6	64	14.6			
Without parents	42	12.3	245	71.6	55	16.1			
Job experience							.912	.006	.002
None	47	12.8	277	75.5	43	11.7			
Part-time	42	12.8	230	70.3	55	16.8			
Full-time	13	14.4	53	58.9	24	26.7			

### Criteria for the Transition to Adulthood

Table 2 shows that the top ten criteria for adult status were: avoid drunk driving, use contraception if sexually active and not trying to conceive a child, avoid illegal drugs, avoid committing petty crimes like vandalism and shop lifting, drive safely and close to speed limit, if a woman become capable of caring for children, avoid becoming drunk, if a man become capable of supporting a family financially, settle into a long-term career, and if a man become capable of keeping a family physically safe. Six criteria belong to Norm compliance, three criteria to Family capacities, and one criterion to Financial independence. The five least endorsed criteria were: have at least one child, married, have obtained driver’s license and can drive an automobile, purchase house, and committed to long-term love relationship. They belong to Family formation and Age-related/biological transitions. The most prevalent categories of criteria were Norm compliance and Family capacities, followed by Financial independence, Age-related/biological transitions, Biological parenthood, and Family formation (see Table 5). Only Norm compliance and Family capacities had means larger than .50 (in a 0-1 scale); standard deviations were rather low (*SD* = .14-.38).

**Table 5:** Means and Standard Deviations of All Variables* for Gender, Age, Living Arrangement, Job Experience, Perceived Adult Status, and the Total Sample (N = 784)

Variables	Men (*n* = 298)	Women (*n* = 486)	17.5-19.9 years (*n* = 477)	20-21.9 years (*n* = 189)	22-27.5 years (*n* = 118)	Living WP (*n* = 437)	Living WOP (*n* = 342)	No job (*n* = 367)	Part-time job (*n* = 327)	Full-time job (*n* = 90)	Non-adults (*n* = 102)	Emerging adults (*n* = 560)	Adults (*n* = 122)	Total (*N* = 784)
	*M* (*SD*)	*M* (*SD*)	*M* (*SD*)	*M* (*SD*)	*M* (*SD*)	*M* (*SD*)	*M* (*SD*)	*M* (*SD*)	*M* (*SD*)	*M* (*SD*)	*M* (*SD*)	*M* (*SD*)	*M* (*SD*)	*M* (*SD*)
Family capacities^a^	.47 (.31)	.56 (29)	.53 (30)	.55 (.31)	.44 (.28)	.52 (.30)	.52 (.30)	.53 (.31)	.52 (.29)	.47 (.30)	.48 (.29)	.53 (.30)	.50 (.31)	.52 (.30)
Norm compliance^a^	.60 (.26)	.72 (.23)	.71 (.23)	.69 (.24)	.52 (.26)	.69 (.25)	.68 (.24)	.70 (.23)	.66 (.25)	.63 (.27)	.65 (.26)	.69 (.24)	.65 (.26)	.68 (.25)
Financial independence^a^	.49 (.30)	.42 (.28)	.42 (.28)	.46 (.29)	.51 (.30)	.44 (.28)	.46 (.29)	.43 (.29)	.46 (.29)	.46 (.26)	.50 (.29)	.45 (.28)	.38 (.28)	.45 (.29)
Family formation^a^	.12 (.22)	.06 (.14)	.07 (.15)	.08 (.19)	.14 (23)	.08 (.18)	.08 (.18)	.08 (.18)	.09 (.18)	.08 (.18)	.09 (.17)	.07 (.16)	.12 (.23)	.08 (.18)
Biological parenthood^a^	.16 (.34)	.19 (.38)	.18 (.38)	.14 (.34)	.21 (.36)	.18 (.37)	.18 (.37)	.18 (.37)	.17 (.36)	.21 (.39)	.15 (.32)	.18 (.37)	.20 (.37)	.18 (.37)
Age-related/ biological transitions^a^	.36 (.23)	.39 (.22)	.38 (.22)	.38 (.24)	.38 (.21)	.37 (.23)	.39 (.22)	.40 (.22)	.35 (.23)	.39 (.22)	.36 (.24)	.38 (.23)	.38 (21)	.38 (.23)
Identity exploration^b^	3.18 (0.58)	3.33 (0.48)	3.33 (0.49)	3.31 (0.47)	2.99 (0.64)	3.25 (0.54)	3.30 (0.51)	3.32 (0.47)	3.30 (0.52)	2.99 (0.66)	3.26 (0.52)	3.31 (0.51)	3.11 (0.57)	3.27 (0.53)
Negativity/ instability^b^	2.48 (0.62)	2.68 (0.66)	2.67 (0.67)	2.54 (0.61)	2.41 (0.60)	2.62 (0.68)	2.58 (0.62)	2.65 (0.66)	2.55 (0.63)	2.60 (0.71)	2.52 (0.69)	2.60 (0.64)	2.66 (0.69)	2.60 (0.65)
Self-focus^b^	3.08 (0.51)	3.11 (0.44)	3.14 (0.45)	3.14 (0.47)	2.90 (0.51)	2.97 (0.46)	3.26 (0.43)	3.16 (0.46)	3.06 (0.46)	3.03 (0.52)	3.01 (0.44)	3.11 (0.47)	3.15 (0.51)	3.10 (0.47)
Experimentation/possibilities^b^	3.19 (0.58)	3.33 (0.47)	3.32 (0.47)	3.25 (0.51)	3.14 (0.68)	3.20 (0.55)	3.37 (0.47)	3.28 (0.50)	3.31 (0.54)	3.11 (0.54)	3.33 (0.49)	3.29 (0.51)	3.17 (0.59)	3.27 (0.52)
Feeling “in-between”^b^	2.91 (0.72)	3.29 (0.69)	3.27 (0.65)	3.13 (0.74)	2.64 (0.77)	3.12 (0.75)	3.17 (0.69)	3.26 (0.65)	3.12 (0.70)	2.75 (0.94)	3.23 (0.72)	3.25 (0.63)	2.56 (0.86)	3.14 (0.73)
Other-focus^b^	2.56 (0.60)	2.56 (0.60)	2.55 (0.60)	2.66 (0.59)	2.56 (0.60)	2.59 (0.58)	2.53 (0.61)	2.57 (0.57)	2.58 (0.62)	2.46 (0.62)	2.39 (0.56)	2.60 (0.60)	2.54 (0.60)	2.56 (0.60)

*Note*. *Except father’s education, for which no statistically significant differences were found.

^a^0-1; ^b^1-4; WP = with parents; WOP = without parents.

### Criteria for the Transition to Adulthood: Differences Among Subgroups

A series of multivariate analyses of variance and covariance were conducted to test for differences in the criteria for the transition to adulthood as a function of gender, age, father’s education, living arrangement, job experience, and perceived adult status.

A multivariate analysis of variance with Bonferroni corrections was conducted to test for gender and age differences in the criteria for the transition to adulthood. A significant multivariate effect was found only for gender, Wilks’ Lambda = .95, *F*(6, 747) = 6.70, *p* < .001, partial η^2^ = .05. Follow-up univariate tests showed significant gender differences in Family capacities, *F*(1, 752) = 6.94, *p* = .009, partial η^2^ = .01, Norm compliance, *F*(1, 752) = 20.10, *p* < .001, partial η^2^ = .03, Financial independence, *F*(1, 752) = 4.06, *p* = .044, partial η^2^ = .01, and Family formation, *F*(1, 752) = 9.92, *p* = .002, partial η^2^ = .01. Women had significantly higher scores on Family capacities and Norm compliance, whereas men had significantly higher scores on Financial independence and Family formation. Although the multivariate effect for age was not significant, follow-up univariate tests showed significant age differences in Norm compliance, *F*(2, 752) = 4.38, *p* = .013, partial η^2^ = .01. Post hoc comparisons (LSD) indicated that the older age group (22-27.5 years) had significantly lower scores on Norm compliance than the younger age group (17.5-19.9 years) (see Table 5). No significant gender x age interaction emerged.

A multivariate analysis of covariance was conducted with Bonferroni corrections to test for perceived adult status differences in the criteria for the transition to adulthood, and with gender and age as covariates. Α significant multivariate effect was found, Wilks’ Lambda = .97, *F*(12, 1496) = 2.11, *p* = .014, partial η^2^ = .02. Follow-up univariate tests showed significant differences only in Financial independence, *F*(2, 753) = 6.88, *p* = .001, partial η^2^ = .02. Post hoc comparisons (LSD) indicated that self-perceived adults had significantly lower scores on Financial independence than self-perceived non-adults and emerging adults (see Table 5).

Multivariate analyses of covariance for father’s education, living arrangement, and job experience yielded no statistically significant results.

### Developmental Features of Emerging Adulthood

Table 3 shows that the five most prominent developmental features of emerging adulthood were: taking responsibility for yourself (Self-focus), planning for the future and defining yourself (Identity exploration), and exploration and many possibilities (Exploration/possibilities). These characteristics were accepted as central features (Agree and Strongly agree combined) by at least 90% of youths. On the contrary, the five attributes which youths considered less characteristic of their lives were: confusion and feeling stressed out (Negativity/instability), self-sufficiency (Self-focus), responsibility for others and commitments to others (Other-focus). They were endorsed by about 50% of participants or less. The most prevalent categories of developmental features were Identity exploration and Experimentation/possibilities, followed by Feeling “in-between”, Self-focus, Negativity/instability, and Other-focus (see Table 5). There was a high agreement among participants with most developmental features (*M* = 2.56-3.27 in a 1-4 scale) and rather low standard deviations (*SD* = 0.21-0.75).

### Developmental Features of Emerging Adulthood: Differences Among Subgroups

A series of multivariate analyses of variance and covariance were conducted to test for differences in the developmental features of emerging adulthood as a function of gender, age, father’s education, living arrangement, job experience, and perceived adult status.

A multivariate analysis of variance with Bonferroni corrections was conducted to test for gender and age differences in the developmental features of emerging adulthood. A significant multivariate effect was found only for age, Wilks’ Lambda = .93, *F*(12, 1460) = 4.19, *p* < .001, partial η^2^ = .03. Follow-up univariate tests showed significant age differences in Identity exploration, *F*(2, 735) = 9.11, *p* < .001, partial η^2^ = .02, Experimentation/possibilities, *F*(2, 735) = 3.12, *p* = .045, partial η^2^ = .01, and Feeling “in-between”, *F*(2, 735) = 14.14, *p* < .001, partial η^2^ = .04. Post hoc comparisons (LSD) indicated that the older age group (22-27.5 years) had significantly lower scores on Identity exploration and Feeling “in-between” than the two younger age groups (17.5-19.9 and 20-21.9 years); for Experimentation/possibilities no statistically significant comparisons were found, although there was a decreasing trend with age (see Table 5). Furthermore, although the multivariate effect for gender was not significant, there was a significant univariate effect on Feeling “in-between”, *F*(1, 735) = 5.37, *p* = .021, partial η^2^ = .01. Women had significantly higher scores on Feeling “in-between” than men (see Table 5). No significant gender x age interaction emerged.

Next, a multivariate analysis of covariance was conducted with Bonferroni corrections to test for living arrangement differences in the developmental features of emerging adulthood, and with gender and age as covariates. A significant multivariate effect was found, Wilks’ Lambda = .88, *F*(6, 727) = 16.19, *p* < .001, partial η^2^ = .12. Follow-up univariate tests showed significant differences as a function of living arrangement in Self-focus, *F*(1, 732) = 75.62, *p* < .001, partial η^2^ = .09, and Experimentation/possibilities, *F*(1, 732) = 18.36, *p* < .001, partial η^2^ = .02. Young people who lived without their parents had significantly higher scores on Self-focus and Experimentation/possibilities than those living with their parents (see Table 5).

To test for job experience differences in the developmental features of emerging adulthood, a multivariate analysis of covariance was conducted with Bonferroni corrections and with gender and age as covariates. Α significant multivariate effect was found, Wilks’ Lambda = .95, *F*(12, 1462) = 2.94, *p* < .001, partial η^2^ = .02. Follow-up univariate tests showed significant differences as a function of job experience in Identity exploration, *F*(2, 736) = 9.22, *p* < .001, partial η^2^ = .02, Experimentation/possibilities, *F*(2, 736) = 3.64, *p* = .027, η^2^ = .01, and Feeling “in-between”, *F*(2, 736) = 7.52, *p* = .001, partial η^2^ = .02. Post hoc comparisons (LSD) indicated that students with full-time job experience had significantly lower scores on Identity exploration, Experimentation/possibilities, and Feeling “in-between” than students with no job or part-time job experience (see Table 5).

A multivariate analysis of covariance was conducted with Bonferroni corrections to test for perceived adult status differences in the developmental features of emerging adulthood, and with gender and age as covariates. Α significant multivariate effect was found, Wilks’ Lambda = .87, *F*(12, 1462) = 8.60, *p* < .001, partial η^2^ = .07. Follow-up univariate tests showed significant differences as a function of perceived adult status for Self-focus, *F*(2, 736) = 4.08, *p* = .017, partial η^2^ = .01, Feeling “in-between”, *F*(2, 736) = 38.54, *p* < .001, partial η^2^ = .10, and Other-focus, *F*(2, 736) = 4.96, *p* = .007, partial η^2^ = .01. Post hoc comparisons (LSD) indicated that self-perceived adults had significantly higher scores on Self-focus than self-perceived non-adults; self-perceived adults had lower scores on Feeling “in-between” than self-perceived non-adults and emerging adults; and self-perceived adults and emerging adults had higher scores on Other-focus than self-perceived non-adults (see Table 5).

Multivariate analyses of covariance for father’s education yielded no statistically significant results.

### Views of the Future

For the question assessing views of overall quality of life in the future, 66.9% of participants responded “better”, 25.8% “about the same”, and 7.3% “worse” than that of their parents. For the question on financial well-being, 39.5% responded “better”, 47.7% “about the same”, and 12.8% “worse”. For the question on career achievements, 61.2% responded “better”, 27.8% “about the same”, and 11.0% “worse”. Finally, for the question on personal relationships, 40.2% responded “better”, 48.7% “about the same”, and 11.1% “worse”.

## Discussion

The purpose of this study was to examine how Greek university students conceptualized criteria for the transition to adulthood and developmental features of emerging adulthood, as well as possible effects of sociodemographic variables and perceived adult status. Optimism for the future was also examined, in an attempt to better understand the transition to adulthood as is currently experienced by studying youth in Greece.

### Perceived Adult Status

More than two thirds (71.4%) of young people in Greece exhibited ambivalence as to their perceived adult status. They felt that they were on the way to adulthood but not there yet (similar results in Petrogiannis, 2011), a finding possibly due to the sociodemographic shifts which have taken place in Greece in recent decades (briefly presented in the Introduction). The proportion found for emerging adulthood in this study is as high as the proportion in the USA (Arnett, 2001; Badger et al., 2006; Nelson & Barry, 2005) and higher than that found in other countries (e.g., Denmark, Israel, Argentina, Czech Republic, China) or subcultures (e.g., ethnic minorities) (Arnett, 2003; Arnett & Padilla-Walker, 2015; Badger et al., 2006; Cheah & Nelson, 2004; Facio & Micocci, 2003; Macek et al., 2007; Mayseless & Scharf, 2003; Nelson et al., 2004, 2012). It appears, then, that the essential developmental feature of emerging adulthood – feeling between adolescence and adulthood – is present in the specific sample of Greek young people.

Larger proportions of women define themselves as emerging adults than men, a finding attributed to strong gender stereotypes still existing in Greece (e.g., compared to women, men are allowed more freedom from parents, are expected to mature earlier and exhibit more independence). This gender difference is consistent with previous research in Greece (Petrogiannis, 2011) and other cultures (Doğan et al., 2015; Seiter & Nelson, 2011). Moreover, the emerging adult status was more prevalent among those students with no job or part-time job experience, whereas the adult status was more frequent among those with full-time job experience, a finding similar to that in other countries (Doğan et al., 2015; Macek et al., 2007).

### Criteria for the Transition to Adulthood

The results of this study show that transition to adulthood is perceived by young people in a complex way. Some items from Independence and Interdependence categories were removed from Arnett’s measure because of low loadings, and a different factor structure emerged from the Greek data. The three most prevalent categories of criteria were Norm compliance, Family capacities, and Financial independence. Contrary to the findings in Western cultures (Arnett, 2001, 2003; Arnett & Padilla-Walker, 2015; Mayseless & Scharf, 2003; Sirsch et al., 2009; Wängqvist & Frisén, 2015), where individualistic criteria were frequently endorsed, in Greece there is an emphasis on more collectivistic markers of adulthood. More specifically, there is a mixture of individualistic and communal criteria, somewhat similar but not identical to that found in Southern Europe (Crocetti & Tagliabue, 2015; Piumatti et al., 2016), Eastern Europe (Nelson, 2009; Vosylis et al., 2015), Turkey (Doğan et al., 2015), and Argentina (Facio & Micocci, 2003), and dissimilar to the findings of the other Greek study (Petrogiannis, 2011), in which criteria denoting Independence were frequently endorsed by university students (although in the latter study no factor analyses were conducted).

In the present study, one of the new factors – Financial independence – may be considered as the individualistic facet of the transition to adulthood in Greece. It is a very interesting, possibly culturally-determined, finding, which reflects Greek young people’s conception of adulthood as a condition stemming from the following accomplishments: finishing education, finding full-time employment, and settling into a long-term career, therefore acquiring financial independence from parents and becoming able to live on one’s own. It was also expressed less frequently from self-perceived adults than all other participants, perhaps because the former felt close to their achievement. This cluster of criteria is a long-standing view of adulthood in Greece and is still evident in the context of severe economic adversity and high youth unemployment rates.

Nevertheless, no longer living with parents, which belongs to this factor, was deemed important by less than one third of young people. This finding, in combination with the low loadings of the excluded criteria denoting independent decision making, establishing equality with parents, and lessening emotional ties with them, indicate that connectedness to parents is typical of youth in Greece (cf. Georgas, Berry, van de Vijver, Κağitçibaşi, & Poortinga, 2006). It is also consistent with the quite high endorsement of Family capacities criteria. More specifically, about two thirds of young people regarded the capabilities to care for children, support the family financially and keep it safe as important indicators of adulthood (similar findings in Petrogiannis, 2011). Thus, in Greece, performing one’s duties towards others while managing to stand on one’s own feet are the main elements of adult status. Besides, compliance to social norms presupposes a degree of self-control, which is typical of young people that have achieved adequate personal autonomy and is also indicative of “psychological adulthood” (Côté, 2000).

The most frequent Norm compliance indicators were “not driving while drunk”, “not having sexual intercourse without protection”, and “not using illegal drugs”. They were deemed necessary for adulthood by more than 80% of the participants and their importance declined with age. About two thirds of participants considered avoiding petty crimes, driving safely, and avoiding drunkenness as important markers. However, avoiding profanity and not having more than one sexual partner were endorsed by only one third of participants. Young people seem to regard as the most typical adult-like behavior all kinds of reckless behavior that do not have negative consequences on others (e.g., avoidance of drunk driving), or on self (e.g., avoidance of illegal drugs), and not behaviors that are indicative of a specific life-style (e.g., number of sexual partners) (see Arnett, 1994, for similar findings and explanations). Avoiding reckless behavior has long been regarded as a crucial sign of adult status (Arnett & Taber, 1994). Non compliance to social norms is a rather frequent phenomenon in Greece and a matter of general concern due to the adverse consequences to fellow citizens (e.g., high frequency of automobile accidents). Similarly, norm compliance was ranked high in Argentina (Facio & Micocci, 2003), a fact attributed by the investigators to the very frequent violation of these norms in their country.

Age-related/biological transitions, as well as specific criteria denoting transitions and belonging to Family formation (e.g., married, have at least one child), were considered necessary for adulthood by rather small percentages of participants. We should note here that less than 1% of them were married or had a child, and that marriage and parenthood are postponed until early thirties in Greece. The majority of external markers of adulthood according to biology, anthropology, sociology, and law were not as important as the internal markers that have a moral quality and cannot be easily assessed. This finding is consistent with other research findings in Greece (Petrogiannis, 2011) and across countries (Arnett, 2001, 2003; Badger et al., 2006; Cheah & Nelson, 2004; Facio & Micocci, 2003; Macek et al., 2007; Mayseless & Scharf, 2003; Nelson & Barry, 2005; Nelson et al., 2004; Piumatti et al., 2016).

Systematic gender differences in the criteria for the transition to adulthood emerged. Consistent with previous research in other countries (Arnett, 2001; Arnett & Padilla-Walker, 2015; Badger et al., 2006; Cheah & Nelson, 2004; Cheah et al., 2010; Doğan et al., 2015; Donoghue & Stein, 2007; Facio & Micocci, 2003; Mayseless & Scharf, 2003; Nelson, 2009; Piumatti et al., 2016; Seiter & Nelson, 2011; Sirsch et al., 2009), women endorsed Family capacities and Norm compliance criteria more frequently than men. In contrast, men rated Financial independence and Family formation criteria as more important for adult status than women. These findings reflect gender socialization processes. Traditionally in Greece, women are socialized to place much importance on family and on the opinion of others, whereas men are reared to be independent and autonomous. Men are expected to have full-time employment because they are still considered as the primary breadwinners of the family. Consequently, according to their views, what enables young men to mature is sexual intercourse, marriage, and parenthood (thus, the high importance assigned to a long-term love relationship).

### Developmental Features of Emerging Adulthood

The developmental features of emerging adulthood were highly prevalent in university students’ lives, according to their own views, thus providing support for Arnett’s formulation. Emerging adulthood appeared to be a time of Identity exploration, Experimentation/possibilities, Feeling “in-between”, Self-focus, Negativity/instability, and Other-focus. The high scores of Identity exploration and Experimentation/possibilities, compared to the other developmental features, are characteristic of most student populations studied in Greece (Leontopoulou et al., 2016) and several contexts (Atak & Çok, 2008; Lisha et al., 2014; Negru, 2012; Sirsch et al., 2009), and are usually higher than in other groups, for example working youth (Crocetti et al., 2015; Doğan et al., 2015; Seiffge-Krenke, 2015).

The slight deviations of the Greek version of IDEA from the original factorial structure may be culturally determined. These deviations appeared mainly in the structure of Self-focus: “time of focusing on oneself” was excluded due to its low factor loading, “time of separating from parents” loaded on Self-focus and not on Identity exploration, and part of the Self-focus structure was a feeling of personal restriction (i.e., “time of feeling restricted”). It seems that, in Greece, Self-focus takes a meaning of personal freedom and independence in the context of the relationships with parents, and less of self-focus per se, and may also entail negative feelings as young people struggle to find their own place in the world. These findings may reflect the dual emphasis on collectivistic and individualistic values, characteristic of the Greek culture (Georgas et al., 2006). This mixed picture also emerged for optimism, which co-existed with the sense of instability, a finding supporting the complex nature of young people’s experience on the way to adulthood and possibly the fact that instability, as a typical feature of emerging adulthood, is not always a pathological state (for a similar explanation see Arnett, 2007).

Whereas in other studies in Greece (Leontopoulou et al., 2016) and other contexts (Atak & Çok, 2008; Lisha et al., 2014) Feeling “in-between” did not emerge as a separate dimension or was not highly prevalent (Hill, Lalji, van Rossum, van der Geest, & Blokland, 2015), in this study it was a distinct and prevalent feature, a finding that supports the main element in the definition of emerging adulthood. Negativity/instability, although present, did not appear to be a prominent feature in university students’ lives (see Hill et al., 2015 for a similar finding). Among the least typical characteristics were confusion and feeling stressed-out, whereas the item on optimism was endorsed by 78% of participants.

Identity exploration, Experimentation/possibilities and Feeling “in-between” were found to decline with age, as expected (for similar findings see Crocetti et al., 2015). Consistently, Feeling “in-between” was least characteristic of self-perceived adults, compared to self-perceived non-adults and emerging adults, whereas Self-focus was most characteristic of self-perceived adults, and Other-focus of self-perceived adults and emerging adults. In accordance with the findings discussed earlier, the dual emphasis on self and on others emerged as a typical feature of self-perceived adulthood.

As for gender differences, Feeling “in-between” was more prevalent in women, a finding consistent with previous research in Greece (Leontopoulou et al., 2016) and other countries (Crocetti et al., 2015; Doğan et al., 2015). No other gender difference was found for the developmental features of emerging adulthood. This is in contrast to other studies, where women scored higher than men on more developmental features (Crocetti et al., 2015; Negru, 2012; Reifman et al., 2007; Sirsch et al., 2009). The gender difference on Feeling “in-between” is congruent with the significantly larger number of self-perceived emerging adults among women than among men. In Greece, women typically experience greater dependency from parents and less freedom compared to men, and this may reinforce women’s ambivalence toward adulthood.

Self-focus and Experimentation/possibilities were more prominent features for university students living without their parents compared to those living with parents, which is a rather expected and easily explained finding (Kins & Beyers, 2010). Interestingly, Identity exploration, Experimentation/possibilities and Feeling “in-between” were less prominent in full-time working youth compared to youth with no or part-time work experience, a finding somewhat similar to that found in Italy and Japan (Crocetti et al., 2015) (there was no significant difference between the two types of living arrangement as a function of job experience). Furthermore, Feeling “in-between”, both as a dimension of the IDEA and as a category of perceived adult status (i.e., emerging adult), was a typical feature of students with no job or part-time job experience, and self-perception as adult was more likely for students with full-time job experience – an expected finding too, similar to that of other studies which compared working to non-working youth (e.g., Doğan et al., 2015; Macek et al., 2007; Seiffge-Krenke, 2015). It is important to note, though, that in the present study the percentage of young people with part-time and full-time job experience combined is higher than 50%, making the sharp distinction between non-working and working youth, or between students and workers, rather artificial – at least as the sample of this study is concerned.

However, we must note that there were no significant differences as a function of father’s education and that many statistically significant differences had a rather small effect size. All these findings suggest that developmental features of emerging adulthood are common among Greek university students, independent of the subgroup they belong to.

### Age of Optimism

There was a cautious optimism about the future among Greek university students. The most optimistic predictions were made for overall quality of life and career achievements, whereas less optimism was expressed about financial well-being and personal relationships. The percentages of optimism found in this study were lower than the ones found in other cultures with the same measure (Arnett, 2000b; Nelson, 2009; Nelson et al., 2004; Seiter & Nelson, 2011). This may be explained by the fact that, at the time of data collection, these young people had already experienced the beginning of the severe financial crisis. These findings, in combination with the large percentage of young people feeling on the way to adulthood but not there yet, and the large percentages considering this period of life as a time of optimism, personal freedom, and open choices as well as a time of restriction, implies that emerging adulthood in Greece is experienced in an intense, heterogeneous, and even ambivalent way. Moreover, the findings provide support for the existence among young people in Greece of a kind of resilient optimism (Arnett, 2012) in the face of socioeconomic adversity.

### Limitations and Suggestions for Future Research

The most notable limitation of this study is the generalizability to other youth populations. The sample, although large and representative of many university departments, was only from Athens, the capital of Greece. There was also an absence of more detailed data on SES and financial constraints of young people and their families, in light of the severe recession in Greece. Moreover, achieved criteria for adulthood were not assessed.

Therefore, a next step in this research field in Greece is to study various subgroups of young people, from different areas of the country and with different educational and job statuses. The role of contextual variables related to the financial crisis, such as unemployement, underemployment, and underpaid jobs, should be examined both cross-sectionally and longitudinally. Also, person-centered approaches (e.g., Tagliabue et al., 2016) may identify clusters or profiles of youth with different conceptions and experiences of the transition to adulthood, by assessing important variables, such as types of identity status or dimensions of identity development, with more detail than in the present research. Τhe interplay of several economic and educational factors may lead to diverse paths to adulthood, at least as far as the felt experience of emerging adulthood, conceptions of what it means to be an adult, and identity outcomes are concerned. Special attention should be paid to Greek young people who are not in education, employment or training (NEET), a subgroup of youth on which little research has been conducted in this country. And, finally, in light of the finding supporting a kind of resilient optimism among young people in Greece, it is useful to examine factors positively influencing young people’s optimistic views about their future despite poor economic conditions, an objective that belongs to a promising field of research, that of the positive emerging adult development (Arnett, 2015).

### Conclusions

Despite these limitations, this study provides clear evidence for the existence of emerging adulthood with its specific developmental features and for the prolonged transition to adulthood in Greece, as far as university students are concerned. Family capacities, Norm compliance, and Financial independence were the major markers of adulthood, indicating a mixture of collectivistic and individualistic views of the transition. Identity exploration, Experimentation/possibilities, and Feeling “in-between” were the most important perceived developmental features of emerging adulthood, and a very high percentage of young people (higher than in many other cultures) felt between adolescence and adulthood. These findings portray a picture of prolonged moratorium (Erikson, 1968) of Greek studying youth, perhaps a forced delay of coming of age (Côté, 2014), possibly explained, at least in part, by existing socio-economic conditions. Systematic gender differences in the conceptions and experiences of emerging adulthood were found, as well as age differences. Interesting significant variations among subgroups also emerged, highlighting important facets of the links between specific contextual factors (i.e., living arrangement, job experience) and transition to adulthood. Finally, on their way to adulthood, university students in Greece were cautiously optimistic about their future.

### Implications for Policy and Practice

The findings of the present study provide useful information for educators, therapists, and policy makers addressing this population both in Greece and in the European context. More specifically, tertiary education in Greece is characterized by restricted mobility opportunities for students (e.g., change of subject of study), which is dissimilar with the educational system of other countries (e.g., USA; Arnett, 2016). This is in sharp contrast to the findings of the present investigation showing that a high percentage of studying youth felt between adolescence and adulthood, and that identity exploration, experimentation/possibilities, and self-focus were highly prevalent in their lives. Thus, the structure of tertiary education in Greece needs to become more flexible and developmentally appropriate, while at the same time providing a solid structure which is highly needed during a less structured and less stable life period compared to adolescence.

Moreover, the findings of the present study support a high degree of ambivalence on the way to adulthood: a large number of self-perceived emerging adults emerged; young people expressed both optimism and instability; whereas many of them endorsed financial independence as a criterion of maturity, they assigned rather little importance to living without parents; and, finally, young people valued both individualistic and collectivistic markers of adulthood. This intersection of positive development and mental health problems (O’Connor et al., 2016) should be taken into account by counselors and therapists when they attempt to promote positive development among youth and to provide mental health services to this population, in a cultural context where family ties seem to remain strong and supportive but may also become a source of stress and strain due to economic adversity.
